# IL-6 in the Ecosystem of Head and Neck Cancer: Possible Therapeutic Perspectives

**DOI:** 10.3390/ijms222011027

**Published:** 2021-10-13

**Authors:** Michal Španko, Karolína Strnadová, Aleš Jan Pavlíček, Pavol Szabo, Ondřej Kodet, Jaroslav Valach, Barbora Dvořánková, Karel Smetana, Lukáš Lacina

**Affiliations:** 1 Institute of Anatomy, First Faculty of Medicine, Charles University, 12800 Prague, Czech Republic; karolina.strnadova@lf1.cuni.cz (K.S.); ales-jan.pavlicek@lf1.cuni.cz (A.J.P.); pavol.szabo@lf1.cuni.cz (P.S.); ondrej.kodet@lf1.cuni.cz (O.K.); barbora.dvorankova@lf1.cuni.cz (B.D.); karel.smetana@lf1.cuni.cz (K.S.J.); lukas.lacina@lf1.cuni.cz (L.L.); 2 Department of Stomatology, First Faculty of Medicine, Charles University and General University Hospital in Prague, 12801 Prague, Czech Republic; jaroslav.valach@lf1.cuni.cz; 3 BIOCEV, Biotechnology and Biomedicine Centre, Academy of Science and Charles University, 25250 Vestec, Czech Republic; 4 Department of Dermatovenereology, First Faculty of Medicine, Charles University and General University Hospital in Prague, 12800 Prague, Czech Republic

**Keywords:** IL-6, head and neck cancer, cancer microenvironment, targeted therapy

## Abstract

Interleukin-6 (IL-6) is a highly potent cytokine involved in multiple biological processes. It was previously reported to play a distinct role in inflammation, autoimmune and psychiatric disorders, ageing and various types of cancer. Furthermore, it is understood that IL-6 and its signaling pathways are substantial players in orchestrating the cancer microenvironment. Thus, they appear to be potential targets in anti-tumor therapy. The aim of this article is to elucidate the role of IL-6 in the tumor ecosystem and to review the possible therapeutic approaches in head and neck cancer.

## 1. Introduction

Head and neck cancer (HNC) is a significant cause of morbidity and mortality worldwide. Its incidence is increasing; approximately 550,000 new cases are diagnosed every year [1]. Head and neck squamous cell carcinoma (HNSCC) is its most frequent histological type [2]. Smoking, alcohol consumption, paan (betel) abuse in some regions and HPV infection are pivotal etiological causes of HNSCC [3]. Furthermore, ageing is substantially involved, as gene-repair mechanisms are reduced after the age of 50. The risk of malignancy is thus further enhanced [4]. In HNSCC, it is alarming that approximately two-thirds of cases are diagnosed at an advanced stage, and roughly 50% of the patients die within two years after the initial diagnosis [5]. Since the number of oncological cases is rising, novel therapeutic approaches need to be developed [6].

The tumor mass is a complex ecosystem. It is formed of cancer cells and a supporting tissue called tumor stroma. It comprises various cell populations (e.g., activated fibroblasts, immune cells, endothelial cells, adipocytes, mesenchymal stem cells) and extracellular matrix [7,8,9,10]. The biological behavior of cancer cells can be significantly modified by stromal cells through intensive intercellular cross-talk. This can be based on their mutual intercellular contacts or paracrine production of bioactive factors [11,12,13].

Cancer-associated fibroblasts (CAFs) produce a plethora of cytokines. Their effect is pleiotropic, i.e., they can modulate functions of various cell types. The function can be either synergistic or antagonistic. Their interplay is a highly complex phenomenon. Chemokines and interleukins are the major cytokines involved in tumor growth and metastatic spread [3].

Interleukin-6 (IL-6) is a crucial component of the cancer microenvironment. It can be produced by cancer cells or their stromal counterparts, e.g., CAFs [10,14,15]. The synthesis and activity of IL-6, though, is not limited to the cells of the cancer ecosystem. It can easily leak out and affect distant tissues and cells. It can also be found in, e.g., hematopoietic cells of bone marrow, epithelial cells, connective tissues and muscle cells [16]. This contributes to the formation of a premetastatic niche. Therefore, IL-6 signaling seems to be a promising target in anti-tumor therapy [6].

Of note, IL-6 is also involved in a plethora of other biological processes such as inflammation, ageing, autoimmune (rheumatoid arthritis) and psychiatric disorders (bipolar disorder and anorexia) [6]. However, it is beyond the scope of this review.

IL-6 is vitally important for many malignant cells; moreover, it is a critical molecule significantly shaping their tumor microenvironment landscape. We believe that this complex mechanism of multifaceted intercellular communication in tumors is worthy of attention. As the IL-6 signaling pathway offers several druggable targets, it was earlier clinically exploited to treat various conditions. We believe that it also holds therapeutic potential for the oncology of solid tumors, including HNC. In this review, we, therefore, aimed to summarize available data on the IL-6 signaling pathway mechanisms and emphasize the IL-6 importance in the context of the tumor microenvironment. We also offer a review of selected therapeutic options available for the purposes of IL-6 pathway regulation.

## 2. IL-6 Signaling Pathway

IL-6 is a glycosylated protein formed of 184 amino acids (MW 23.7 kDa) [17]. It was originally described to stimulate B lymphocytes and hepatocytes [18]. It is synthesized by multiple cell types including cancer and stromal cells in many types of human malignancies [16,17].

There are two principal pathways of IL-6 signaling. The first, referred to as classic signaling, utilizes the membrane-bound receptor (IL-6R). It is expressed only on hepatocytes and certain types of leukocytes [19,20]. The signal is subsequently transduced by membrane-bound α-receptor glycoprotein 130 (gp130), which initiates a cascade of intracellular processes. The second pathway, named trans-signaling, is activated by the binding of IL-6 to the soluble form of IL-6R (sIL-6R). sIL-6R is produced by proteolytic cleavage (shedding) of IL-6R from the cell membrane by metalloproteases ADAM10 and ADAM17 [21,22]. Of note, human sIL-6R can also be a product of alternative splicing of IL-6R mRNA. However, this mechanism is of minor significance [23]. The trans-signaling pathway can activate any cells since gp130 is expressed ubiquitously. It primarily involves pro-inflammatory reactions, whilst the classic signaling is dedicated to anti-inflammatory and regenerative processes [6,21]. IL-6R also appears to be pro-cancerogenic [24]. The soluble form of gp130 (sgp130), which is most likely formed by alternative splicing, is known to inhibit the trans-signaling pathway naturally [21,25].

As mentioned above, gp130 is the inceptive step of intracellular signaling. There are three possible consequent pathways: JAK-STAT (Janus kinase and signal transducer and activator of transcription) pathway, MAPK (mitogen-activated protein kinase) cascade and PI3K (phosphatidylinositol-3-kinase) cascade [22,26]. The cross-talk between these signaling units still requires detailed elucidation [22].

Interestingly, the cytokine receptors lack intrinsic kinase activity [22]. Janus kinases (JAK) are associated with the cytoplasmic part of gp130. Once IL-6 is bound to IL-6R, JAK kinases are activated by autophosphorylation. Of note, there is a family of JAK kinases associated with IL-6 signaling: JAK1, JAK2 and Tyk2. JAK1 is, though, the essential type. The activation of JAKs causes phosphorylation of tyrosine motifs located on the cytoplasmic part of gp130. The four most distal phosphorylated motifs (Y767, Y814, Y905 and Y915) bind STAT1 and STAT3 monomers. They are consequently phosphorylated within their tyrosine sites (STAT1: Y701, STAT3: Y705), dimerized and translocated into the nucleus [22,26,27]. This traditional description, though, appears to be simplified. Recent studies have revealed that STATs can dimerize even without cytokine stimulation and tyrosine phosphorylation [28,29,30,31]. The pre-dimerization step requires the N-terminal region of the protein, whereas the cytokine-dependent dimerization requires tyrosine Y705 phosphorylation [32]. Even the translocation of STAT3 into the nucleus is not strictly based upon phosphorylation. It requires the N-terminal domain. In fact, regardless of cytokine stimulation, non-phosphorylated STATs can transfer from the cytoplasm to the nucleus and vice versa [32]. However, IL-6-dependent tyrosine phosphorylation increases the pool of STAT3 in the nucleus [33]. Such a finding corresponds to the original concept of JAK-STAT signaling.

The nuclear activity of STAT3 engages the CBP/p300 transcriptional adaptor. Its function is to regulate transcription and the course of the cell cycle by histone acetylation [22,34]. Moreover, it acetylates STAT3 on both N-terminal (lysines 49 and 87) [35] and C-terminal domains (lysine 685) [36]. Such a modification enhances the interaction between STAT3 and CBP. Thus, STAT3-dependent gene expression is potentiated [35,36,37]. On the other hand, STAT1 signaling is considered to be of minor significance [22].

**Figure 1:**
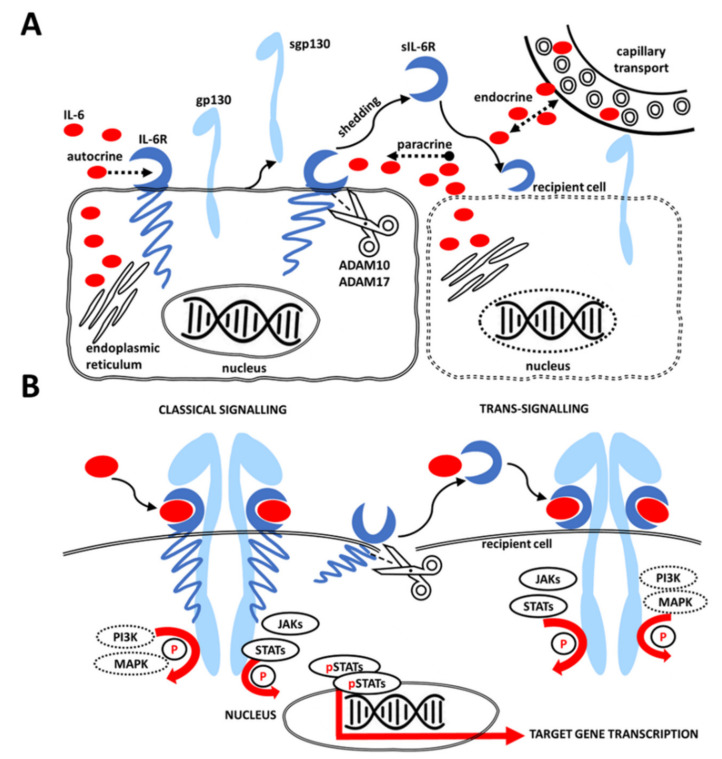
(**A**): All cells express signal transducer glycoprotein gp130. This molecule can be membrane-bound (gp130) or soluble (sgp130). Expression of IL-6 and its receptor, IL-6R, can be cell-type restricted. IL-6R, if expressed, can be bound to the membrane or it can be released as a soluble receptor (sIL-6R) due to the protease activity (by ADAM10 or ADAM 17). Once released from the membrane (shedding), it can also reach cells that do not express their own IL-6R. The IL-6 cytokine can act on the cell of the same origin (autocrine effect), on the neighboring cells (paracrine effect) or even exert systemic effects of endocrine nature (it is transported via the blood stream into capillaries). (**B**): Activation of gp130 by the IL-6/IL-6R complex (classic signaling) or by IL-6/sIL-6R (trans-signaling) leads to tyrosine phosphorylation (“P”) of transcription factors JAKs/STATs, PI3K and MAPK. Upon their phosphorylation, the regulatory molecules transmit the signal downstream, translocate into the nucleus and bind to enhancers of IL-6-type cytokine target genes.

Apart from JAK-STAT signaling, IL-6 can initiate the MAPK (mitogen-activated protein kinase) cascade and the PI3K (phosphatidylinositol-3-kinase) cascade. The cytoplasmic tyrosine-759 motif of gp130 is responsible for a balance between the STAT3 and MAPK cascades. Such an equilibrium is essential for the organism’s homeostasis [38,39] and its disturbance may lead to the development of inflammatory and proliferative diseases [40,41]. STAT3 regulates expression of cyclins D2, D3 and A, phosphatase cdc24A and cell cycle inhibitors p21 and p27 [39]; activated MAPK is a crucial mitogenic impulse [38]. Hyperactivity of both STAT3 and MAPK correlates with splenomegaly and thrombocytosis. In contrast, diminished activity of STAT3 and MAPK is associated with reduced acute-phase response and thrombocytopenia. Attenuated phosphorylation of the tyrosine-759 motif increases IL-6-dependent STAT3 activity [42,43] and reduces MAPK activity [44,45], which is related to gastric adenomas, autoimmune arthritis and increased Th1 cytokine expression. Conversely, phosphorylation of the tyrosine-759 motif reduces STAT3 activity and potentiates MAPK activity. This leads to reduced acute-phase gene expression and increased Th2 cytokine expression [46] (Figure 1).

STAT3 can be inactivated through binding of JAK feedback inhibitor SOCS3 to the phosphorylated tyrosine-759 motif in gp130. The activation of MAPK is based on the binding of the adapter protein and tyrosine phosphatase SHP2 to the same tyrosine-759 motif [38,44,45,47,48]. Both of these proteins utilize SH2 domains for binding to gp130 [49]. Additionally, phosphorylation of the tyrosine-759 motif of gp130 leads to recruitment of protein Gab1 [50]. Its function is to connect MAPK and PI3K with the IL-6 receptor complex [51].

IL-6 signaling can be suppressed in multiple ways. As previously mentioned, SOCS3 and SHP2 bind the phosphorylated 759-tyrosine motif and inhibit JAK-STAT signaling [47]. The STAT-independent signaling (MAPK, PI3K) is thus initiated. IL-6 signaling can also be regulated by the receptor complex internalization and its subsequent degradation [52,53]. This happens both constitutively and by IL-6R activation. Of note, cytokines such as IL-1α and TNFα also contribute to receptor internalization [54]. Eventually, activated STAT dimers can be negatively regulated by PIAS (protein inhibitor of activated STAT). PIAS3 is known to interfere with the phosphorylated tyrosine-705 domain of STAT3 [55].

## 3. IL-6 in the Context of Head and Neck Cancer

### 3.1. Tumor Microenvironment

Despite the recent advances in clinical medicine, head and neck carcinoma remains a significant cause of morbidity and mortality worldwide [1]. The limited efficacy of conventional anti-tumor therapy originates in the eradication of quickly dividing cancer cells [56,57]. In contrast, slowly proliferating progenitors and stem cells remain relatively spared and protected by a surrounding niche called tumor microenvironment (TME) [58]. In the search for new therapeutic approaches, IL-6 appears to be a promising target since it is a potent player within the TME. A number of adverse cancer-associated effects are related to IL-6 signaling [6].

The tumor microenvironment is a very complex structure. The cancer cells are surrounded by a variety of supporting cell types (e.g., cancer-associated fibroblasts, immune cells, endothelial cells, adipocytes, mesenchymal stem cells) and extracellular matrix [7,8,9,10]. All types of these cells produce IL-6 [59]. Notably, the stromal cells are not just quiet bystanders—they are substantially involved in the tumor growth regulation [6,60].

IL-6 signaling is complex and is dependent on the available signal (IL-6 cytokine), receptor equipment (IL-6R) and signal transducer gp130. Glycoprotein gp130 is present ubiquitously on all cell types. In contrast, cytokine production and receptor expression are more restricted. Interleukin can act directly on the cell in an autocrine manner. It can also influence the neighboring cells in the vicinity of the cytokine source in a paracrine manner. However, IL-6 can also enter the circulation and act at long distances, and eve exert systemic effects of endocrine nature. 

Such versatile arrangement of cytokine production and release along with receptor mobility in the soluble form is a prerequisite for precise orchestration of the tumor cells and their supporting stromal population into one malignant machinery. One cell type can easily support and foster the other components in case of a lack of any component of the receptor complex. In this view, it is also possible that the membrane-bound IL-6/IL-6R complexes on transmitter cells activate the gp130 receptors on neighboring receiver cells [61]. Moreover, such versatility justifies the concept of cluster signaling [62], where some cells are able to trans-present IL-6 through the complex containing IL-6R bound on their membrane and thus interact with gp130 expressed on another cell in intimate proximity [63].

Having said all that, IL-6 seems to fit very well to the concept of the tumor microenvironment. Should the intention of targeting IL-6 become a successful therapeutic strategy in oncology one day, it is inevitable that the structure and rules of the tissue microenvironment must be observed. 

### 3.2. Cancer-Associated Fibroblasts (CAFs)

Immune cells were the first recognized producers of IL-6. However, CAFs are also known to be a potent source of IL-6 in the context of the tumor microenvironment. We will focus on them in detail because they have been extensively studied in recent years [64]. CAFs represent the most abundant cell type in the tumor stroma and secrete many regulatory molecules—growth factors, cytokines and immune modulators [65]. They can be readily recruited from the local fibroblasts. Besides that, CAFs can also to some extent arise from bone marrow mesenchymal cells, pericytes, endothelial cells and smooth muscle cells [60]. Although CAFs represent a heterogeneous group of cells [66], conserved expression of α-smooth muscle actin (α-SMA), vimentin, tenascin C, fibroblast-specific protein-1, fibroblast-activating protein and neural-glial protein is observed [65,67]. CAFs play an important role in tumorigenesis, local invasiveness, tumor angiogenesis and chemoresistance. CAFs can induce epithelial-mesenchymal transition (EMT) and thus facilitate distant metastasizing [68]. It has been reported that fibroblasts isolated from squamous cell carcinoma and basal cell carcinoma significantly differ from normal fibroblasts, since their phenotype is shifted towards epithelial cells [69,70,71,72].

There are numerous studies describing remarkable similarities between a tumor and a healing wound [10,15,73,74]. Fibroblasts are substantially involved in the formation of both tumor stroma and granulation tissue [13,71,74,75]. Fibroblasts can transform into myofibroblasts and hence modulate wound contraction [76]. Myofibroblasts also influence the biological behavior of the tumor [75]. Wound healing is a cascade of precisely regulated events. It comprises blood clotting, inflammation, proliferation and maturation with consequent remodeling [58]. All these steps are also precisely orchestrated in terms of their timing. If temporal and spatial regulation fails, wound healing is suboptimal. Of note, the granulation tissue, product of the proliferative phase of normal healing, resembles the tumor stroma.

Fibroblasts embedded in these tissues readily produce not only structural molecules of ECM, but also multiple cytokines and growth factors, and thus potentiate angiogenesis and epithelial cell proliferation [77,78,79,80]. This can lead to rapid reepithelization of the wounded area in normal healing tissue. However, in cancer, identical stimuli will result in increased tumor mass. The pathways are shared; the result is given by the current tissue context. According to whole-genome transcriptome profiling, certain cytokines and ECM proteins have similar regulatory roles in both tumor growth and wound healing—namely, IL-6, IL-8, chemokine (C-X-C domain) ligand 1, galectin-1, and fibronectin [15]. Interestingly, basal and squamous cell carcinomas were referred to as caricatures of a healing skin wound [58].

The cancer field theory was initially coined on the background of oral cancer; however, it was later applied to models of many cancer types [81]. It was primarily proposed to describe the wide impact of cancerogenic genotoxic agents affecting epithelial cells and consequently triggering neoplastic growth in some of them. However, these harmful stimuli also inevitably impact the surrounding fibroblasts. Therefore, the precise diagnostic distinction of normal or abnormal cells should not be applied to cancerous cells only, but should also be extended to the broader landscape forming the tumor-supportive microenvironment [10,13]. These cells, including CAFs, are per se not genetically malignant, but their activity in the tissue is at least tumor-promoting [82]. Therefore, the control of their key signaling mechanisms could have therapeutic implications.

### 3.3. Effects of IL-6 Expression

**Figure 2:**
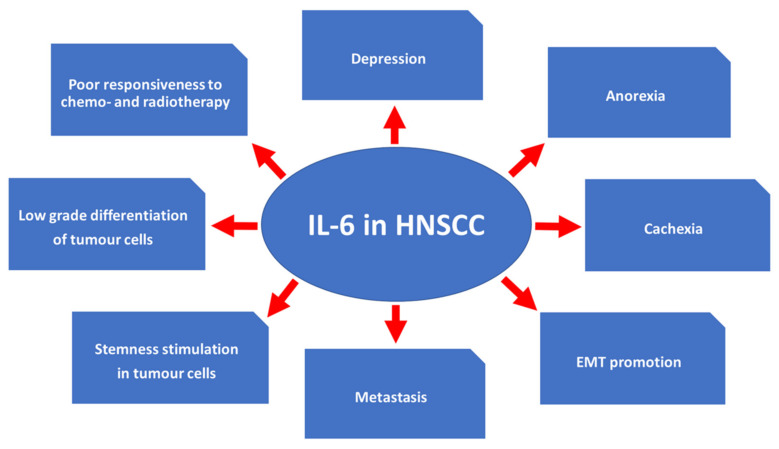
Effects of IL-6 expression in HNSCC [6,15,83,84,85,86,88,89,90]. The role of the IL-6 cytokine is multifaceted in HNSCC. In the brain, IL-6 can be linked to the onset of depression and can also cause food intake disorders (anorexia), leading to wasting (cachexia). At the cellular level, IL-6 promotes epithelial-to-mesenchymal transition, which leads to the formation of metastatic lesions. Maintenance of low differentiation is associated with certain stemness features in tumor cells, predisposing to a poor response to chemo- and radiotherapy. However, many of these factors can further potentiate each other interdependently.

IL-6 is understood to modulate the tumor microenvironment significantly. It likely keeps the low-grade differentiation of cells in squamous cell carcinoma [15] and promotes their epithelial-mesenchymal transition (EMT) [83]. IL-6 produced by different cell types in the cancer microenvironment stimulates migration of cancer stem cells, hence the metastatic potential increases [84,85]. Overexpression of IL-6 indicates a poor response to chemo- [86,87] and radiotherapy [86,88,89]. Eventually, an elevated level of IL-6 may contribute to bone metastasizing [90]. IL-6 also affects the metabolism of oncological patients by induction of cancer cachexia and afflicts the central nervous system, where it may induce anorexia and depression (Figure 2) [6].

### 3.4. Serum and Salivary Concentrations of IL-6 in HNSCC and Their Potential Role in Diagnostics

The concentration of IL-6 in the serum and other biological fluids is increased in HNSCC patients [91,92,93,94]. The physiological serum level of IL-6 is lower than 10 pg/mL [95]. Riedel et al. reported higher serum IL-6 concentrations in HNSCC patients compared to healthy controls [96]. In contrast, a different study by Andersson et al. did not prove a statistically significant difference between HNSCC patients and healthy individuals in terms of serum IL-6 concentrations [97]. It should be noted here that many HNSCC cases are primarily exophytic tumors. Therefore, an increased level of IL-6 in the serum might not be an optimal biomarker of early disease. However, this does not diminish its critical importance during the progression and metastasis formation.

Oral squamous cell carcinoma (OSCC), a topographic subtype of HNSCC, was also reported to have elevated serum IL-6 concentrations [94,98]. There is robust evidence that elevated IL-6 expression correlates with tumor progression and consequently also with lower patient overall survival [85,86,87,89,91,96,98,99,100,101,102,103,104,105,106]. In detail, increased IL-6 levels were associated with increased tumor size and the presence of nodal metastasis in OSCC patients [99]. Another study also reported higher T-stage, clinical stage, bone invasion and tumor depths in OSCC [98]. HNSCC patients with T3/T4-stage, positive nodal metastases and advanced stage were also found to have their IL-6 levels elevated [96,105,106].

We can conclude that IL-6 seems to be a candidate biomarker related to the overall- and disease-free survival in OSCC patients [98]. Its value could be further increased in combination with other biomarkers. Such multiplexing can further facilitate diagnostic interpretation. For instance, overexpression of both IL-6 and nuclear myoferlin indicates more dismal survival rates when compared with isolated myoferlin overexpression [107]. A similar result can be found when there is overexpression of both IL-6 and protein arginine methyltransferase-5 [108]. Also, analyzing the serum IL-6 concentration jointly with salivary IL-8 concentration can improve the sensitivity and specificity [109].

Of note, increased systemic availability of IL-6 can have rather unexpected implications. Human behavior is extremely complex, and it is challenging to apply principles of scientific reductionism strictly to it and follow the increase of a single cytokine. However, it is worth mentioning that circulating IL-6 has a clinically documented impact on several behavioral aspects. All of these behavioral patterns might have health-related consequences. Duffy et al. analyzed the possible relationship between health-related behavior and IL-6 levels in patients prior to HNSCC treatment. The results showed that particular addictive behavior (represented by smoking status), weak sleep scores, higher age, and more advanced tumor stage were significantly associated with increased serum IL-6 concentrations. Interestingly, tumor location, alcohol consumption, physical activity, body mass index, as well as ethnicity, education and financial status had no relevant correlation with serum IL-6 levels [110]. We can only speculate regarding the causality of these factors. 

Likely, the early disease stages may not be fully represented by biomarkers detected in circulating body fluids. More proximal samples could overcome this issue in localized disease. Therefore, saliva has attracted the interest of researchers during recent years [111,112,113]. It is sometimes referred to as “liquid biopsy”. A non-invasive procedure is required for sample collection, sampling is cost-effective and cancer cells are located within its vicinity [114]. Hence, saliva seems to be a valuable substrate for the early detection of pathological conditions of the oral cavity [112,115].

The physiological salivary level of IL-6 oscillates around 10 pg/mL [116]. Significantly higher concentrations have been reported in OSCC patients compared to healthy individuals [92,117,118,119,120,121,122,123,124,125]. Moreover, some studies showed that OSCC had significantly higher IL-6 levels than oral potentially malignant disorders (OPMD; e.g., leukoplakia, erythroplakia, lichen planus, oral submucous fibrosis) [116,119,123,124,125,126]. Notably, it was also higher compared to chronic oral inflammatory diseases (e.g., chronic periodontitis) [123].

OSCC and these premalignant conditions are sometimes difficult to treat radically by surgery. This increases the importance of consequent watchful follow-up. A sensitive biomarker of early recurrence would thus be of very great diagnostic value. IL-6 concentrations dropped significantly after the successful treatment of OSCC. Salivary IL-6 was therefore proposed as a useful marker to monitor the treatment response [127] and to screen the locoregional recurrence during the patient’s follow up [128,129]. However, it is necessary to associate the laboratory finding with the clinical status of the patient. Of note (as of mid-2021), there are no regulatory authority-approved salivary diagnostic tests available in the USA or Europe for evaluating the risk of oral diseases, including head and neck cancer.

### 3.5. Anti-IL-6 Therapy

Anti-IL-6 targeting is a promising therapeutic approach in many ways, since activation of IL-6 signaling is associated, for instance, with aggressive tumor growth [99], metastasizing [84,85] and poor responsiveness to chemo- and radiotherapy [86]. A beneficial effect of the blockade can also be increased if used in combinations. Such benefit was observed when IL-6 was targeted together with TNF-α. It is known that TNF-α, similarly to IL-6, is engaged in the occurrence of cancer cachexia and depression [6,130,131]. There are other reports suggesting that a simultaneous blockade of IL-6 and IL-8 can further potentiate the therapeutic outcome [15,132,133,134].

IL-6 can be produced at the tumor site by various cells, but it can also leak and be active in remote body parts. The presence of tumor-associated cytokines in systemic circulation can be a critically important signal for the establishment of premetastatic niche [135], the prerequisite for circulating malignant cell harboring and consequent metastasis formation [10]. Deprivation of tumor cells from these supportive and protective stimuli provided by the microenvironment leaves malignant cells more vulnerable. Therefore, control over IL-6 production could be a clinically attractive strategy. Of note, cytokine synthesis can be inhibited without affecting the viability of cells. This suggests good tolerance and safety profile of this approach and makes this strategy convenient for therapeutic combinations.

Moreover, a plethora of drugs for lowering IL-6 production and release have recently become available on the market and are ready for therapeutic repurposing. The list of these drugs includes well-known anti-inflammatory substances such as acetylsalicylic acid (Aspirin), indomethacin, corticoids, steroidal pregnanes or statins [136,137,138].

On the background of the COVID-19 pandemic, this topic has attracted much attention recently [139]. IL-6 is a multifactorial cytokine and plays a central role in the acute inflammatory response resulting in cytokine storm, a condition which might be fatal for some patients [140]. Upon this ground, several old drugs were extensively investigated for their potential benefits in severe COVID-19 infection. Particularly great attention was dedicated to antimalarials, namely chloroquine and hydroxychloroquine. Based on the recently available Cochrane database analysis, the outcomes of this treatment are controversial [141]. Despite recently strong and polarized opinions on antimalarials among scientists, politicians and the general public, we have to highlight that these substances successfully stood the test of time and were widely used as treatment of several serious autoimmune conditions such as lupus and rheumatoid arthritis. There are evidence-based indications for antimalarials and scientifically sound data suggesting that antimalarials can lower the production of IL-6 in lupus [142] and arthritis [143] patients.

Mechanistically, the deregulated immunity of autoimmune conditions and smoldering protumorigenic inflammation observed in cancer [144] share multiple important features and regulatory molecules, including IL-6 [59]. The immune deregulation of cytokine storm is a very different concept of immune reaction. Failure of antimalarials in acute conditions does not diminish their potential value in chronic inflammation. However, the data on the application of antimalarials in cancer is somewhat sparse and requires careful attention in the future. There are recently running clinical trials focusing on the beneficial role of antimalarials (namely derivatives of artemisinin) in hepatocellular cancer (NCT02304289). A commercially available antimalarial drug, hydroxychloroquine, was used in clinical trials focusing on brain tumors, breast cancer, lung cancer, melanoma, myeloma and other malignancies [145]. For head and neck cancer, a promising effect of quinacrine in combination with platinum derivatives was reported in mouse models [146]. Collectively, antimalarials could potentially exert effects on cell invasion, chemotaxis, cellular trans-differentiation, and clonogenicity in cancer; they are also inhibitors of autophagy [147].

However, when targeting IL-6 production, it is worthy of notice that there are also important physiological functions of this molecule that should not be suppressed entirely. IL-6 is also, e.g., a prominent myokine regulating the homeostasis of skeletal muscles [148]. Here, significant elimination of IL-6 synthesis could have deleterious effects. This function of myokine is of autocrine or paracrine nature.

Therefore, targeting of released and circulating IL-6 represents another therapeutic concept. The first experience with the therapeutic use of anti-IL-6 antibodies dates back to 1988, when mouse anti-IL-6 was tested in patients with multiple myeloma [149]. In the second approved indication, anti-IL-6 was used in patients with Castleman’s disease, where overproduction of IL-6 from the germinal centers of hyperplastic lymph nodes was described [150]. In recent years, the manufacturing of monoclonal antibodies in large quantities for therapeutic purposes has become an affordable option for the pharma industry. It is also possible to produce chimeric or even achieve fully humanized antibodies [151]. These antibodies bind to and neutralize human IL-6 directly, thus decreasing the levels of available free IL-6, and prevent binding to its receptor. For this purpose, siltuximab (CNTO 328) as a chimeric, human-murine immunoglobulin monoclonal antibody produced in Chinese hamster ovary cells was introduced to the market. Later, it was followed by sirukumab (also known as CNTO136), which is a human anti-interleukin (IL)-6 immunoglobulin G1 kappa (IgG1k) monoclonal antibody (mAb), and clazakizumab [152]. For routine use in rheumatoid arthritis approval, additional studies were requested to further define the safety profile of sirukumab [152].

Clazakizumab (in clinical trials also described as ALD518 and BMS-945429), humanized rabbit monoclonal antibody, was raised against interleukin-6 and suggested for treatment of musculoskeletal aspects of psoriatic arthritis and recently also for the management of Late Antibody-Mediated Kidney Transplant Rejection [153].

Olokizumab was also developed for the treatment of rheumatoid arthritis [154]. Mechanistically, it inhibits binding between the IL-6/6R complex and signal transducer gp130. Concerning the availability of IL-6, this offers a particular therapeutic advantage, because this blockade increases systemic levels of available free IL-6 to a lesser degree than other monoclonal antibodies preventing binding of IL-6 and IL-6R (as discussed below).

Full humanization seems critically essential in these applications, because administration of any biologics with immunogenic potential can elicit immune responses leading to the consequent loss of their therapeutic effect. However, this seems to be relatively rare in recently available biologics.

Strategically, targeting the redundantly produced cytokine via antibodies might be a rather labor-intensive, costly and sometimes ungrateful effort. Concerning the ratio of available cytokine/receptor molecules, it seems more prudent and favorable to focus the therapeutic intervention on the IL-6 receptor side [155].

The anti-IL-6R biologics can target either extracellular or intracellular signaling components of the receptor. It is more demanding to administer the drugs within the intracellular compartment since the structures are less accessible [22]. Additionally, downstream effectors, namely JAKs and STATs, could also be targeted. However, it is worthy of notice that these messengers are activated (via their phosphorylation) by a broader range of cytokines, not exclusively by IL-6 [27]. Therefore, we touch them only briefly in our review; however, we have included them in Tables—if their development has reached clinically relevant stage. Similarly, we have deliberately decided to limit the extent of our attention dedicated to highly experimental approaches using gene editing, microRNAs, gene knockouts of signaling cascade members, etc., because these are recently far from clinical applicability [63].

As indicated above, probably the most significant experience with IL-6 blockade was gained in the treatment of rheumatic diseases, especially rheumatoid arthritis (RA), using an antibody against the IL-6 receptor, Tocilizumab [156].

Tocilizumab recognizes the IL-6-binding site of the human IL-6 receptor and thus inhibits IL-6 signaling through competitive blockade of IL-6 binding. Regular dosing of tocilizumab in patients causes no antibody-dependent cellular cytotoxicity or complement-dependent cytotoxicity in cells expressing IL-6R. Tocilizumab recognizes both IL-6R on the cell membrane and soluble IL-6R circulating in body fluids and inhibits both IL-6 signaling and trans-signaling.

Tocilizumab improved clinical signs and symptoms of RA, laboratory parameters, radiological manifestations and ameliorated RA’s effects on patient-reported outcomes, activities of daily living and quality of life when administered as monotherapy or in combination with conventional synthetic disease-modifying antirheumatic drugs [157]. A relatively fundamental study of tumor biology was also presented in clinical trials studying the anti-proliferative effect of Tocilizumab in non-small cell lung cancer cells [158]. Alraouji and coworkers described the effect of Tocilizumab in combination with cisplatin in triple-negative breast cancer. In this study, Tocilizumab potentiated toxicity for cancer stem cells [159]. Thus, tumor stem cells can be a source of self-renewal of tumor clones and failure of conventional chemotherapeutics. The introduction of checkpoint inhibitors into tumor therapy was completely revolutionary. Cemiplimab, as an anti-PD-1 antibody, has shown an excellent therapeutic response in patients with advanced cutaneous squamous cell carcinoma, and it is only a matter of time before it is introduced into HNSCC therapy [160,161]. However, in some patients, there is a weak or no therapeutic response to checkpoint inhibitors. In addition, the immune-mediated mechanisms of resistance have been shown to be challenging to overcome. IL-6 may be one of the molecules involved in the processes of this resistance. It is evidenced by a study of blocking IL-6 and anti-PD-1 simultaneously. Higher levels of IL-6 were detected in some melanoma patients treated with anti-PD-1, which was also associated with a worse prognosis. A simultaneous blockade of IL-6 and this signaling led to an increase in the expression of INF-γ produced by CD4^+^ T cells and consequent enhancement of anti-PD-1 therapy in a mouse model of melanoma [162]. Insufficient expression of INF-γ in the tumor microenvironment is also considered a mechanism of resistance to checkpoint inhibitors.

Tocilizumab was the “first on the market” representative of this class. It was followed in later years by sarilumab with comparable therapeutic outcomes [163]. Very recently, Satralizumab, a humanized IgG2 monoclonal antibody binding soluble and membrane-bound human IL-6R, was approved for autoimmune optic neuromyelitis [164].

Similarly to IL-6 neutralizing antibodies, there are potential risks associated with the immunogenicity of IL-6R-blocking antibodies. However, these occur rarely in the case of tocilizumab.

Another important issue is the stability of serum concentrations after parenteral administration. The half-life of tocilizumab is approximately two weeks; however, in case of olokizumab, it is around one month. This parameter can be modified, e.g., by engineering using an Fc region mutation to prolong the half-life, as proved by Gerilimzumab, a humanized llama antibody with femtomolar potency [165].

To improve this important pharmacological parameter even further, Vobarilizumab (ALX-0061) was designed as an anti-IL-6R nanobody linked to the anti-human serum albumin domain [166]. Bispecific peptide nanobodies represent single-domain antibody fragments that combine the advantages of conventional biologics with some of the features of small-molecule drugs. A nanobody is much smaller than conventional immunoglobulin (conventional IgG monoclonal antibody MW is 144–148 kDa versus nanobody MW of 26 kDa). The small size improves bioavailability; albumin binding provides increased in vivo half-life of the therapeutic molecule.

It has been extensively studied whether IL-6R can also be inhibited to some extent by small drug inhibitors. A smaller molecule can be easily administered, e.g., orally, which can offer better comfort to the patient. Unlike in cases of e.g., CXCLR1/2 or TGFBRI/II, surprisingly we do not have any highly selective small drug inhibitors of IL-6R available currently.

As mentioned earlier, the IL-6 signaling uses glycoprotein gp130 as a signal transducer. Therefore, this molecule represents an attractive therapeutic target. It is expressed by virtually all cell types, which further increases its value. Attempts to provide therapeutic blockade via monoclonal antibodies raised against gp130 were published already in the 1990s [167]. Surprisingly, different antibodies were able to antagonize the biological activities of all cytokines belonging to the IL-6 cytokine family in a specific manner.

For many years, the chemical synthesis of gp130 inhibitors was thought to be extremely challenging. This gave gp130 the reputation of a conventionally undruggable target. However, some natural compounds can bind to the extracellular domain of gp130, namely Madindoline-A. The binding is specific and non-covalent, and displays relatively low affinity [168]. Synthetic analogues of Madindoline-A with improved pharmacologic parameters were synthesized later [168].

Using strategies of molecular design, potent inhibitors were finally synthesized in the last decade—namely SC144 [169], LMT-28 [170].

More recently, it was discovered that bazedoxifene and raloxifene and their derivatives, routinely used drugs for postmenopausal osteoporosis in clinics, inhibit IL-6/gp130 protein–protein interactions. By this mechanism, drugs of the bazedoxifene type and their derivatives cause significant inhibition of IL-6-dependent STAT3 phosphorylation [171]. Recent data suggested that even these well-known drugs can be repurposed for cancer treatment. The proof of this concept was confirmed in gastrointestinal cancer in a mouse model [172].

For signal transduction, gp130 is typically bound to the cell membrane. However, the soluble gp130 form is also known. The mechanism leading to the production of soluble gp130 has been discussed; whether it is formed via alternative splicing, or as suggested more recently, is a product of BACE cleavage [173]. In any case, the soluble form of gp130 binds the IL-6/IL-6R complex, but does not transduce the signal to the cell anymore. This can also be understood as an inhibitory effect, specifically inhibiting trans-signaling. Of note, trans-signaling inhibitor olamkicept was well tolerated in the treatment of inflammatory bowel disease, and this drug was administered without signs of immunosuppression in patients [174]. Gp130 is a large molecule, but it could be manufactured in a miniaturized form, which could be a pharmacologically important modification [173].

Multiple preclinical studies on IL-6 targeting have been completed (see Table 1) [63,88,175,176,177,178,179,180,181,182,183,184,185,186,187,188,189,190,191,192,193,194,195,196,197,198,199,200,201,202,203,204,205,206,207,208,209,210,211]. All focused both on the production of IL-6 and its signaling. There is an array of therapeutics possibly applicable to HNSCC treatment. They were tested either in vitro or in vivo; a few of the studies combined both approaches. Interestingly, some of the investigated therapeutics are already commercially available for the treatment of autoimmune diseases (e.g., Tocilizumab in rheumatoid arthritis [16]). Generally, state of the art anti-IL-6 compounds interfere with IL-6 production, IL-6 availability, inhibition of IL-6R or the IL-6/sIL-6R complex, inhibition of IL-6R signal transducer gp130 and downstream signaling effector molecules [22]. As mentioned above, the results are predominantly preclinical.

On the other hand, there is a rapidly increasing number of approved IL-6-related therapeutic agents, which have not been tested in HNSCC yet (see Supplementary Table S1). As these drugs have already acquired approval and are marketed for other indications, this also offers certain hopes for HNSCC. It is known very well that drug repurposing has a number of interrelated advantages, such as simplification in the regulatory procedures for introducing a previously approved drug on the market. These medications are already well characterized in terms of the drug’s safety and toxicity. This makes the process considerably faster, and therefore also cheaper compared to de novo drug discovery. Hence, robust clinical trials need to be conducted in order to verify the experimental conclusions in clinical practice.

## 4. Conclusions

HNSCC remains a challenging field of medicine because of the high incidence and unfavorable result of therapy in numerous patients. In many cases, standard surgical and oncological therapy fails. There is a critical need for novel therapeutic approaches. IL-6, as a highly potent cytokine, appears to be a promising target. The concept of IL-6 targeting was proved in rheumatologic diseases.

However, several issues relating to IL-6 biology remain unanswered. It is unclear why IL-6 signal inhibition leads to clinically significant benefits for patients with some, but not all, rheumatologic conditions.

Tumors can have different underlying genetic causes, which can result in disease heterogeneity. Due to this heterogeneity, any therapy can be effective in only a subset of the patient population. Such inherent variability of cancer requires increased diagnostic precision of reliable biomarkers and personalized approaches to therapy.

If IL-6 is rightfully believed to be a biologically powerful player in cancer biology, here comes an inevitable question: Why has IL-6 not gained a similar position as a recognized biomarker (of any cancer type including HNSCC) so far? We believe that IL-6 and IL-6-related signaling differ greatly from the routinely used biomarkers in cancer research. More conventional biomarkers are based mostly on malignant cell properties in terms of DNA mutation, RNA expression, protein presence or release, etc. This simplistic approach centered on a malignant cell might be misleading in the case of IL-6. The IL-6 signaling mechanism is far more complex and exceeds a single cell type. It cannot be assigned to the malignant cell population only. It requires a broader view, and one must take the whole tumor microenvironment into account, as was also proposed for HNSCC in our previous review [13]. It is not critical whether a particular cell type expresses the cytokine or the receptor, because the other components of the tumor ecosystem can readily substitute for whatever is missing. The cytokine can diffuse and reach areas remote from where it was produced. Moreover, the soluble receptor from donor cells can activate acceptor cells that do not express it on their own. Thus, IL-6 signaling deregulation is more likely a marker of overall sick social liaisons of the tumor microenvironment rather than a tool for the precise assessment of individual members of this ecosystem, which was also demonstrated for HNSCC in vitro and in clinical material [15]. This is highly challenging for routine analytic methods such as immunohistochemistry. Approaches based on multiplexing could be very helpful in this in the future.

Of note, individualization of therapy can lead to unsustainable expenses in healthcare systems. Therefore, a careful selection of targets applicable to broader patient populations can be beneficial. IL-6 is an essential component in the cancer microenvironment orchestration and contributes to some of the adverse effects of the malignant disease (e.g., cachexia and depression).

A plethora of potential anti-IL-6 therapeutics have been preclinically investigated; many available drugs could also be repurposed for application in cancer therapy (shortlisted in Supplementary Table S1; [25,146,154,155,165,169,170,173,174,228,229,230,231,232,233,234,235,236,237,238,239,240,241,242,243,244,245,246,247,248,249,250,251,252,253,254]). Available data indicate potential benefits for certain types of solid malignancies, which could also apply to HNSCC patients. However, there is still a need for validation of these preliminary data in sufficiently large clinical studies that could finally prove the efficacy and safety of this newly emerging therapeutic concept.

## Figures and Tables

**Table 1:** List of recently tested anti-IL-6 therapeutics.

Agent	Mode of Action	Tested in HNSCC			Approved for Human Application in (Primary Indication):
		In Vitro	In Vivo (Animal Study)	Clinical Trial (Registered on Clinicaltrials.gov)	
2-O-Methylmagnolol	◼ downstream signaling inhibition	Wang et al., 2018 [207]	Wang et al., 2018 [207]	-	-
AMD3100 (CXCR4 inhibitor)	**✼** inhibition of IL-6 production	Tang et al., 2008 [187]	-	-	-
PD98059 (MEK inhibitor)	**✼** inhibition of IL-6 production	Tang et al., 2008 [187]	-	-	-
Ammonium pyrrolidinecarbodithioate (PDTC)	**✼** inhibition of IL-6 production	Tang et al., 2008 [187]	-	-	-
Aryl hydrocarbon receptor antagonist (BAY 2416964)	**✼** inhibition of IL-6 production	DiNatale et al., 2011, 2012 [185,186]	-	NCT04069026 § [212]	-
Bazedoxifene	◆ gp130 blockade	Yadav et al., 2017 [209]	Yadav et al., 2017 [209]	-	Postmenopausal osteoporosis [213]
Cepharanthin	**✼** inhibition of IL-6 production	Tamatani et al., 2007 [180]	-	-	-
Cilengitide (EMD 121974) + cetuximab	**✼** inhibition of IL-6 production	Wichmann et al., 2017 [177]	-	-	Cetuximab–metastatic colorectal cancer, advanced HNSCC [214]
Curcumin	**✼** inhibition of IL-6 production ◼ downstream signaling inhibition	Chakravarti et al., 2006 [184] Cohen et al., 2009 [183] Meyer et al., 2011 [210]	Yu et al., 2013 [63]	NCT04208334; Thambamroong et al., 2016 [215]	Dietary supplement [216]
Celecoxib + Simvastatin	**✼** inhibition of IL-6 production	Gehrke et al. 2017 [208]	-	-	Celecoxib - NSAID (non-steroidal anti-inflammatory drug) [217] Simvastatin - reduction of LDL (low-density lipoprotein) cholesterol blood levels [218]
Cyclopentenone prostaglandin 15d-PGJ2	◼ downstream signaling inhibition	Siavash et al., 2004 [188]	-	-	-
Epigallocatechin gallate	◼ downstream signaling inhibition	Lin et al., 2012 [182]	-	-	Dietary supplement [219]
Guggulsterone	◼ downstream signaling inhibition	Macha et al., 2011 [181] Leeman-Neill et al. [220]	Leeman-Neill et al. [220]	-	Dietary supplement [220]
Honokiol	**✼** inhibition of IL-6 production ◼ downstream signaling inhibition	Chang et al., 2018 [206]	-	-	Dietary supplement [221]
Lactoferrin (Human, recombinant)	**✼** inhibition of IL-6 production	Wolf et al. [178]	Wolf et al. [178]	-	-
RhoC knockdown (lentiviral vector-based shRNA)	**✼** inhibition of IL-6 production	Islam et al. [193]	-	-	-
L-leucine-methylester (lysosomotropic agent)	**✼** inhibition of IL-6 production	Kross et al. [199]	-	-	-
Luteolin	◼ downstream signaling inhibition	Tu et al. [179]	-	-	Dietary supplement, popular in chinese traditional medicine [222]
MEDI5117 (anti-IL-6 humanized monoclonal antibody, IgG1, with Enhanced Serum Half-Life; also known as WBP216)	**♢** IL-6 neutralization	Finkel et al., 2016 [195]	Finkel et al., 2016 [195]	-	-
P276-00 (cyclin-dependent kinase inhibitor)	**✼** inhibition of IL-6 production	Mishra et al., 2013 [200]	Mishra et al., 2013 [200]	-	-
Rapamycin	**✼** inhibition of IL-6 production	Ekshyyan et al., 2016 [196]	-	-	Immunosuppressive drug [223]
SB203580 (p38 MAPK inhibitor)	**✼** inhibition of IL-6 production	Riebe et al., 2007 [197] Jing et al., 2016 [198]	Jing et al., 2016 [198]	-	-
Siltuximab (anti-IL-6 chimeric (human-murine) monoclonal recombinant antibody (IgG1κ))	**♢** IL-6 neutralization	-	-	NCT00841191; Angevin et al., 2014 [224,225]	Management of systemic inflammation in COVID-19 [226]
Insulin-like growth factor-II mRNA binding protein-3 and podoplanin knockdown (lentiviral vector-based shRNA)	**✼** inhibition of IL-6 production	Hwang et al., 2016 [194]	Hwang et al., 2016 [194]	-	-
TLR-9- knockdown (siRNAs)	**✼** inhibition of IL-6 production	Ruan et al., 2014 [211]	-	-	-
Tristetraprolin knockdown (lentiviral vector-based shRNA)	**✼** inhibition of IL-6 production	Van Tubergen et al., 2011, 2013 [203,204]	Van Tubergen et al., 2013 [204]	-	-
Tetrathiomolybdate	**✼** inhibition of IL-6 production	Teknos et al., 2005 [202]	Teknos et al., 2005 [202]	-	-
Tocilizumab (anti-IL-6R humanised IgG1 monoclonal recombinant antibody )	❖ IL-6R blockade	Matsuoka et al., 2016 [88] Stanam et al., 2015 [190] Shinriki et al., 2009, 2011 [191,192]	Poth et al., 2010 [189] Stanam et al., 2015 [190] Shinriki et al., 2009, 2011 [191,192]	-	Rheumatoid arthritis [227] Giant cell arteritis [227] Polyarticular Juvenile idiopathic arthritis [227] Systemic Juvenile Idiopathic arthritis [227] Cytokine Release syndrome [227] Management of systemic inflammation in COVID-19 [226]
Triazolothiadiazine	◆ gp130 blockade	Sen et al., 2017 [201]	-	-	-
Oxazole-piperazine	◆ gp130 blockade	Sen et al., 2017 [201]			-
WP1066 (JAK inhibitor)	◼ Downstream signaling inhibition	Zhou et al., 2014 [205]	Zhou et al., 2014 [205]	-	-
YM529 (third-generation bisphosphonate, Minodronic acid)	**✼** inhibition of IL-6 production	-	Cui et al., 2005 [176]	-	-

§ ongoing clinical trial, **✼** IL-6 production, **♢** IL-6 neutralization, ❖ IL-6R blocking, ◆ gp130 blocking, ◼ downstream signaling inhibition.

## Supplementary Materials

The following are available online at https://www.mdpi.com/article/10.3390/ijms222011027/s1.

## Abbreviations

Aside from official gene names, the following abbreviations were used in the text and legends:

α-SMA	α-Smooth muscle actin
CAF	Cancer-associated fibroblast
CBP	CREB-binding protein
ECM	Extracellular matrix
EMT	Epithelial to mesenchymal transition
GAB1	GRB2-associated-binding protein
gp130	Β-Receptor glycoprotein 130
HNC	Head and neck cancer
HNSCC	Head and neck squamous cell carcinoma
HPV	Human papilloma virus
IFN	Interferon
IL-6	Interleukin 6
IL-8	Interleukin 8
IL-6R	Receptor for IL-6
JAK	Janus kinase
JAK-STAT	Janus kinase and signal transducer and activator of transcription
MAPK	Mitogen-activated protein kinase
OPMD	Oral potentially malignant disorder
OSCC	Oral squamous cell carcinoma
PIAS	Protein inhibitor of activated STAT
PI3K	Phosphatidylinositol-3-kinase
RA	Rheumatoid arthritis
SOCS3	Suppressor of cytokine signaling 3
STAT	Signal transducer and activator of transcription
TME	Tumor microenvironment
TNF-α	Tumor necrosis factor α
