# Supplementary material for: IL-6 in the Ecosystem of Head and Neck Cancer: Possible Therapeutic Perspectives
Source: Int J Mol Sci. 2021 Oct 13;22(20):11027. doi: 10.3390/ijms222011027 (PMC8540903; doi:10.3390/ijms222011027)
Supplement: Supplementary file 1 [file ijms-22-11027-s001.zip › ijms-1320431-supplementary.pdf]

**Supplementary Table S1. List of anti-IL-6 drugs (not tested in HNSCC)**

| Antibodies and Biologics Targeting IL-6 Signaling (not Tested in HNSCC) | | | | |
|---|---|---|---|---|
| **Agent** | **Mechanism** | **Trade name** | **Description** | **Approved for Clinical Application** |
| Vobarilizumab (ALX-0061) [228,252] | ❖ IL-6R blocking | - | nanobody (antibody fragment) | - |
| Sarilumab [228–230] | ❖ IL-6R blocking | Kevzara | human monoclonal antibody | Rheumatoid arthritis |
| Satralizumab [231] | ❖ IL-6R blocking | Enspryng | humanized monoclonal antibody | Neuromyelitis optica spectrum disorder (NMOSD) |
| Clazakizumab [232] (CSL300, ALD518 or BMS945429) | ◊ IL-6 neutralization | - | humanized monoclonal antibody | - |
| Olokizumab [233,253] | ◊ IL-6 neutralization (and prevents binding to gp130) | Artlegia | human monoclonal antibody | Rheumatoid arthritis (only in Russia) |
| Sirukumab (CNTO136) [234] | ◊ IL-6 neutralization | - | human monoclonal antibody | - |
| Olamkicept [174] (FE 999301, FE301, TJ301) | ◆ gp130 blocking | - | recombinant human fusion protein (gp130 extracellular domains dimerized by the Fc part of human IgG1) | - |
| Soluble gp130 complete or miniaturized [25,173] | ■ Downstream signaling inhibition | - | soluble gp130 (miniaturized, chimeric) | - |
| Elsilimomab (B-E8) [155] | ◊ IL-6 neutralization | - | murine monoclonal antibody | - |
| B-R6 [155] | ❖ IL-6R blocking | - | human monoclonal antibody | - |
| B-R3 [155] | ◆ gp130 blocking | - | human monoclonal antibody | - |
| Gerilimzumab [165] (RYI 008) | ◊ IL-6 neutralization | - | llama humanized antibody | - |

| | | | | |
|---|---|---|---|---|
| Baricitinib [235,254] | ■ Downstream signaling inhibition | Olumiant | JAK inhibitor | Rheumatoid arthritis<br>Atopic dermatitis<br>Covid-19 (in emergency use) [254] |
| Ruxolitinib [235,236] | ■ Downstream signaling inhibition | Jakafi | JAK inhibitor | Myelofibrosis<br>Polycythemia vera<br>Steroid-refractory acute graft-versus-host disease |
| Tofacitinib [235,237] | ■ Downstream signaling inhibition | Xeljanz | JAK inhibitor | Rheumatoid arthritis<br>Psoriatic arthritis<br>Ulcerative colitis |

**Other drugs targeting IL-6 signaling (not tested in HNSCC)**

| Agent | Mechanism | Trade name | Description | Approved for clinical application |
|---|---|---|---|---|
| Chloroquine [238–240] | ✻ IL-6 production | Aralen | antimalarial drug | Malaria<br>Extraintestinal amebiasis |
| Hydroxychloroquine [241,242] | ✻ IL-6 production | Plaquenil | antimalarial drug | Malaria<br>Discoid and systemic lupus erythematosus<br>Rheumatoid arthritis<br>Covid-19 |
| Artemisinin (and derivatives) [243,244] | ✻ IL-6 production | Artesunate | antimalarial drug | Malaria |
| LLL12B [245] | ■ Downstream signaling inhibition | - | STAT3 inhibitor | - |
| Raloxifene [246,247] | ■ Downstream signaling inhibition | Evista | selective estrogen receptor modulator | Postmenopausal osteoporosis |
| Madindoline-A (and synthetic analogues [248,249] | ◆ gp130 blocking | - | gp130 inhibitor | - |
| SC144 [169] | ◆ gp130 blocking | - | gp130 inhibitor | - |
| LMT-28 [170] | ◆ gp130 blocking | - | gp130 inhibitor | - |

| | | | | |
|---|---|---|---|---|
| Quinacrine [250,255], in combination with platinum derivatives) [146] | ■ Downstream signaling inhibition | Mepacrine, Atabrine | Quinacrine - antimalarial drug<br>Platinum derivatives - chemotherapeutics | Giardiasis<br>Rheumatoid arthritis<br>Systemic lupus erythematosus<br>Pneumothorax |
| Steroidal pregnanes (plant derived—glaucogenin C and hirundigenin) [251] | ✻ IL-6 production | - | phytocompounds with anti-inflammatory activity | - |

✻ IL-6 production, ◊ IL-6 neutralization, ❖ IL-6R blocking, ◆ gp130 blocking, ■ Downstream signaling inhibition
